# A Method for Modeling the Individual Convenient Zone of a Human

**DOI:** 10.3390/ijerph191610405

**Published:** 2022-08-21

**Authors:** Bogdan Branowski, Marek Zabłocki, Przemysław Kurczewski, Maciej Sydor

**Affiliations:** 1 Institute of Transport, Faculty of Civil and Transport Engineering, Poznan University of Technology, 60-965 Poznań, Poland; 2 Department of Woodworking and Fundamentals of Machine Design, Faculty of Forestry and Wood Technology, Poznań University of Life Sciences, 60-637 Poznań, Poland

**Keywords:** upper limb range of motion, physical strength, ergonomics, digital human model, participatory design, user-centered design, furniture design, senior, persons with disabilities

## Abstract

When designing products to fit a specific user, it is essential to know the user’s upper limb range and strength capabilities at each point of the range space. This is particularly relevant when those capabilities are atypical, e.g., in cases of nonstandard body dimensions, disability, or old age. In this paper, we describe a new method to measure and model the strength capabilities at each point of any person’s upper limb range and then present this information in the form of an Individual Convenient Zone (ICZ) model, which is helpful in virtual product prototyping (CAD) for a specific user. The proposed new method includes creating a database of multiple, detailed, spatial-force characteristics, quickly identifying and modeling the ICZ of any human, and analyzing the ergonomics of a product using a digital human model in combination with the ICZ model. The paper also describes an example of how the proposed methodology can be used to customize kitchen furniture design to the ICZ of a specific senior. The expected result of incorporating ICZ into the design is a better fit between the designed product and the user’s needs, supporting user-centered design methodology. Using ICZ enables the involvement of end-users in product design (participatory design). This is particularly important when designing for people with mobility impairments who are more sensitive to nonergonomic solutions. The ICZ modeling method described in this article may have broader applications beyond kitchen furniture design; it could be used to design workspaces and other similar areas where humans reside and perform manipulation activities.

## 1. Introduction

The dimensions of the human body, the ranges of motion in the joints, and muscular strength determine the range space of the upper limbs and the amount of force exerted on objects at any point in the range space. We named this range space the “individual convenient zone” (ICZ). Within this space, it is possible to quickly and conveniently apply physical forces to objects, i.e., manipulate them. The ICZ is heterogeneous in terms of strength/physical strength ability, e.g., the farther the hand is from the body the lower the possible lifting force is (directed upwards). The ICZ dimensions and the available manipulation forces change with a change in body position, for example, they are different when sitting or standing. ICZ parameters are vital in situations in which a person remains in the same position and wants to reach something as comfortably as possible. This is particularly the case when the person is, for example, a senior who has difficulty walking and therefore prefers to stand in one place to prepare a meal in the kitchen; or is a nondisabled person who performs manipulative work and does not want to waste time moving repeatedly. In both cases, it would be convenient for them to have everything they need within the range of their upper limbs and for heavier and more frequently used items to be located closer to hand. This can be achieved by fitting the dimensional furniture arrangement (topology) to the ICZ. It is therefore helpful to know the spatial and strength characteristics of the person that will be using the specific furniture layout.

Accordingly, the problem addressed in this paper can be generalized: how can the available manipulation space of a specific individual be correctly modeled so that the model can be used in product design? A product unsuited to a person will almost certainly have functional deficiencies and may even be harmful to health or hazardous to use. This issue is fundamental if the prospective user, who will choose the product dimensions according to their needs, is a person with nonstandard needs, e.g., a person with a locomotor disability or a senior. Data on people with nonstandard needs are not available in anthropometric and strength atlases. People with limited dexterity are more sensitive to nonergonomic solutions because they are less able to adapt to products that are not dimensionally adapted to their anthropometric dimensions and strength capabilities.

Most studies on strength exercise have been carried out using standard postures. The measured results for numerous and very diverse people have been averaged and provided in the form of recommended strength ranges in standards or textbooks. For example, the scientific literature provides information on strength during pushing and pulling in a standing position [1,2,3]. The summarized and processed information on maximum possible strengths for different action directions can be found in the so-called biomechanical atlases or standards [4,5,6]. Such data are helpful for universal design, i.e., designing products comfortable for everyone or a particular group of people, with defined parameters. However, averaged strength values from measurements in standardized positions may not have predictive accuracy; they may not be suitable for predicting the optimal topology of products designed for a person with atypical needs.

It is recognized that [7] studied the exertion of strength in the free-standing position and found a high recurrence of mean strength in the examined group of subjects; however, they investigated the motor skills of a relatively homogeneous group of college students. Predicting actual strength values at different points of the upper limb range is challenging, and modeling is subject to errors [8]. In atlases of human measurements, e.g., [9,10,11], averaged data are provided to meet the requirements of typical adult users (such atlases use statistically averaged data from a few model individuals, so-called probands, who correspond to the centiles given: 5, 50, and 95). These data do not consider groups of users with significantly different strength abilities due to disabilities or atypical body dimensions, so the data from atlases rarely correspond to the dimensions and strength abilities of a specific individual. Using these data, it is possible to design universally but not in an entirely customized way.

A standard method currently used to incorporate human anthropometric dimensions into a designed product is CAD modeling using digital human models (DHM). Several models are described in the literature and examples of commonly used DHMs are JACK, Human Builder, and RAMSIS [12], which are offered as standalone programs or modules in well-known CAD programs. For example, SiemensNX uses JACK, Catia uses Human Builder, and RAMSIS is widely used in car interior design [13,14]. With built-in databases, DHMs enable the design of a product tailored dimensionally to a person of selected body dimensions or tailored to a larger group of people within the desired size range, analogous to anthropometric atlases. Some DHMs permit the calculation of available strength for a limited number of body positions at an indicated point in the manipulation space. However, such an analysis could be subject to substantial error for a user with unknown and atypical upper limb range and strength capabilities, e.g., a senior or a person with impaired mobility.

Taking into account the following assumptions that:

- For people with unusual needs, particularly seniors, people with disabilities, or others who significantly deviate in their abilities from the average 50th percentile human, it is difficult to obtain reliable and complete anthropometric data in combination with strength data;
- There are cases in which a precise dimensional product adaptation to the, “individual convenient zone” (ICZ) of a specific user is justified. Modern product design and production technology make this possible, provided that the anthropometric and strength characteristics of the user are known.

It is justified to say that when designing it is advisable to consider each person’s individual ranges and strengths, particularly for the atypical person. However, this raises concern that the measurement of these characteristics will be complicated, lengthy, will require expertise on the part of the researcher, or that the application of the measurement data will be inefficient, e.g., overly complicating the product design. 

The idea of modeling the ICZ and how this can be measured for design purposes was the subject of two previous publications by the authors of this article [15,16]. The procedure proposed in this article concerning the generation and utilization of data for product design is a new and beneficial approach to creating usable spaces that are well suited to humans. Our paper aims to describe an innovative methodology for modeling the ICZ. Section 2 presents this new method, and Section 3 illustrates the use of this method to design a kitchen space tailored to a specific senior.

## 2. Proposed Methodology for Easy Modeling of the “Individual Convenient Zone”

### 2.1. The Concept of Modeling the Convenient Zone of a Human and Adapting Product Dimensions to This Zone

The methodology proposed in this paper for modeling the individual convenient zone (ICZ) of a particular individual and customizing product dimensions to this zone consists of three problem areas:

- Problem area 1. Creation of a database of spatial and strength characteristics based on the laboratory measurements of persons with as much variation as possible in body dimensions and strength capabilities (repository of ICZ models);
- Problem area 2. Simplified measurement and categorization of spatial and strength characteristics of a particular individual and the elicitation of the optimal ICZ model for that individual from the database. The measurement of spatial and strength characteristics is carried out in the most simplified way possible, and the categorization and selection of the appropriate ICZ model are carried out automatically, based on an algorithm;
- Problem area 3. Ergonomic analysis of the designed product, i.e., the correctness of the virtual model design of the product, is evaluated using the digital human model (DHM) containing the anthropometric data of a given person supplemented with the ICZ model.

**Figure 1:**
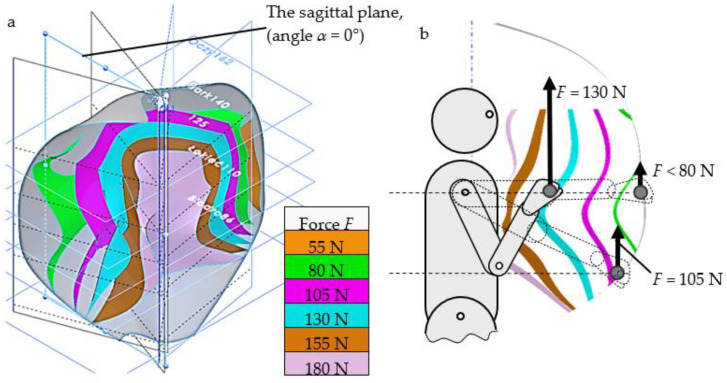
Example model of the individual convenient zone (ICZ) of a particular human, describing range and strength variations of one upper limb (outermost layer represents the range in grey, colored layers indicate the strength values that this person can exert upwards): (**a**) Geometric form of strengths and ranges in axonometric projection; (**b**) dimensions of the range zone and strength variations of the upper limb in the sagittal plane (*α* = 0°).

Problem area 1 requires a detailed measurement of anthropometric dimensions (in compliance with ISO 20685-2 [17]) and the newly proposed spatial and strength characteristics of as many people as possible of varying height (corresponding to the limb range) and differing strength capabilities (corresponding to different force exertion capabilities at different points of the upper limb range). The selection of individuals for laboratory testing should be subject to the criteria outlined in the standards, e.g., ISO 15537 [18]. The spatial and strength characteristics measured represent the upper limb ranges and the variable strength values within these ranges. To create the database, each person’s raw measurement data (a cloud of points with assigned strength values) are transformed into constant and strength spaces according to the approximation and interpolation of the inverse function mapping model, as described in the literature [16]. Following the laboratory measurements and processing, a repository of individual convenient zone models is created, describing the strengths and dimensional characteristics of the individuals. The measuring station for laboratory measurements of human spatial and strength attributes has been patented, and its principles of operation are presented in two patent documents [19,20]. Figure 1 shows examples of spatial and strength characteristics corresponding to the range of manipulation and variability of upper limb strength determined for a specific individual. These characteristics constitute the human ICZ model.

Reliably measuring the complete spatial and strength characteristics of each individual tested is fairly complicated and requires up to several hundred measurements (e.g., taking *F*-force measurements at 100 points throughout the range space). The time taken to perform such measurements is relatively long and requires the supervision of specialists, e.g., physiotherapists, to ensure the safety of the measurement process and the necessary breaks between strength measurements. However, it is possible to enter known strength values from the literature into such a database and use laboratory tests to fill any gaps in the data.

Problem area 2. This involves using the rapid measurement method for the range and variation of the upper limb strengths of a specific individual. The objective is to quickly identify the spatial and strength characteristics of the person being measured and assign them to a ready-made model from the database. The rapid measurement method aims to “decipher” the appropriate, simplified set of spatial and strength data that identifies a specific individual but does not form a complete dataset of the spatial distribution and ranges of generated strength (i.e., they do not form an ICZ).

**Figure 2:**
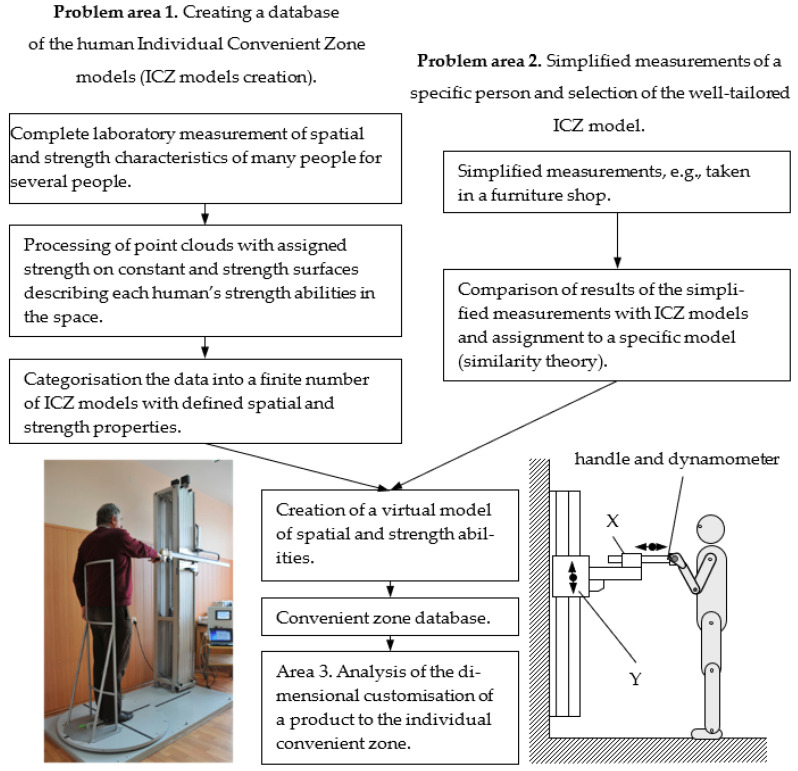
Algorithm of procedure for measuring convenience.

Figure 2 presents the links between the described problem areas 1 and 2. Pictured to the right of Figure 2 is a test rig for laboratory testing of range zone dimensions and human strength values (building the ICZ repository). In contrast, the left-hand side of Figure 2 shows a test rig for simplified measurements (addressing problem area 2, a simplified approach to measurement and classification, permitting the allocation of the corresponding ICZ model from the repository).

The data from the simplified measurements should be compared at selected points in space with the data from the database (repository of Individual Convenient Zones). This comparison will enable selection of the optimal model, i.e., the most similar in all parameters to the person’s characteristics captured via the simplified measurements, from a database of individual convenience zone models.

### 2.2. Determining the Similarity of Spatial and Strength Characteristics of the User of the Designed Technical Object with a Predetermined Person Examined

A search is conducted for the similarity between the *A_b_* features of the individual convenient zone models in the database with the results of a simplified study, i.e., the simplified model with *A_u_* features. Various similarity calculation methods described in the literature can be used [21,22,23]. These methods permit the calculation of both the similarity and dissimilarity (distinction) between objects. The complete model results from laboratory tests (*A_b_*), and the simplified model is derived from simplified measurements (*A_u_*) as a set of coordinates with space and strength capabilities assigned to these coordinates. A measure of the similarity of the models is the distances between the points and the differences in the strength values assigned to these points. Therefore, model similarity can be represented as a parameter reflecting the number and strength of the relationships between the two models being compared.

Simplified measurements (identification of *A_u_* model features) include measurements of five selected human dimensional and strength characteristics:

- *D*_1*u*_—dimensions of the upper limb range space in the sagittal plane (*α =* 0°), at shoulder height;
- *F*_1*u*_—upper limb strength at maximum range within the plane (*α* = 0°), at shoulder height;
- *F*_2*u*_—upper limb strength at maximum range within the plane (*α* = 60°), at shoulder height;
- *F*_3*u*_—upper limb strength at midrange (*D*/2) within the plane *α* = 0°, at shoulder height;
- *F*_4*u*_—upper limb strength at the maximum range within the plane *α* = 0°, at elbow height.

**Figure 3:**
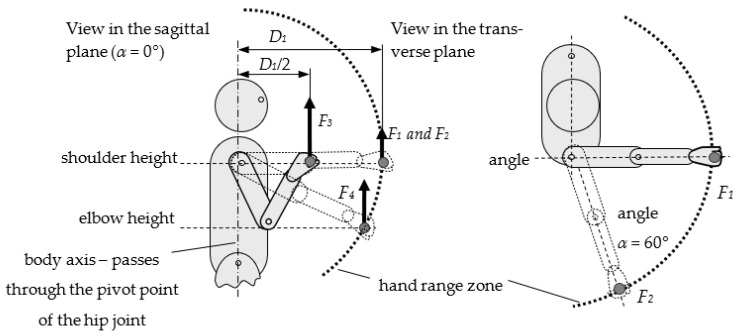
Measuring points during simplified measurements and *A_u_* model determination (explanations: *F*_1*n*_, the strength of the upper limb at the full range *D*_1_ at shoulder height (*α* = 0°); *F*_2*n*_, the strength of the upper limb at the full range *D*_1_ at shoulder height for (*α* = 60°); *F*_3*n*_, the strength of the upper limb at midrange *D*_1*n*_/2 at shoulder height (*α* = 0°); *F*_4*n*_, the strength of the upper limb at full range *D*_1_ at elbow height (*α* = 0°); *D*_1_, arm range within the plane *α* = 0° at shoulder height; *D*_1*n*_/2, arm midrange within the plane *α* = 0° at shoulder height.

This can be entered as $A_{u}=\wedge_{n=1}^{5}F_{u,n}\cup D_{u,1}$. These features are shown in Figure 3.

Two premises influenced the choice of features for determining the simplified *A_u_* model: (1) These parameters are easy to measure for any human being (e.g., in a furniture showroom), and (2) the selected manipulation ranges are frequently used when performing manipulation activities. However, the choice of these features may differ depending on needs. To create the simplified *A_u_* model, it is advisable to use additional and easily accessible features that will improve the fit of the models. These include height, shoulder height, and elbow height.

It was assumed that all features measured in the simplified measurements have an equal impact on the similarity of comparable convenient zone models; hence, all the features are of equal importance in the calculations. Obtaining an optimal match between the features of the Au and Ab models makes it possible to generate a reasonably faithful model of an individual user’s convenient zone without conducting expensive and time-consuming laboratory tests. The probability of obtaining a high degree of similarity between the convenient zone model and the actual spatial and strength characteristics of each person is dependent on the size of the database (the more extensive the database, the better) and the number of features compared in both models (the more features compared, the better).

The comparison uses methodical measures of similarity sim(*x*) (i.e., object proximity) or distance (i.e., object distance). The similarity and distance dimensions are presented equivalently and can be transformed:

(1) $$\mathrm{sim}\left(x\right)=\frac{\sum_{i=1}^{n}{|a}_{ib}{-a}_{iu}|}{n}$$

where: *i* is the number of features used for comparison. When sim(*x*) = 1 the objects are entirely different and when sim(*x*) = 0 the objects are entirely similar.

Before entering data for the similarity calculation, the coordinates of the points should be normalized (e.g., Z score) relative to a fixed reference point (using the interval of the normalized variable *R = A_i_* _max_ − *A_i_* _min_). The result of comparing each pair of features is transformed to the set <1;0>. By applying the zero standard score method, the *a_i_* value is calculated from the relationship:

(2) $$a_{i}=\frac{A_{i}\;{-\;A}_{i\;\min}}{A_{i\;\max}\;{-\;A}_{i\;\min}}$$

where: *A_i_* is the trait under study, *A_i_*_max_ and *A_i_*_min_ are successively the maximum and minimum values found in the set *A_i_* for the *i*-th feature.

The result of comparing each pair of traits refers to each of the traits, $a_{i}=\wedge_{i=1}^{5}Fa_{u,\;i}\cup d_{u,1}$ where *f*_1_*–f*_4_ and *d*_1_ are the 0–1 normalized equivalents of traits *F*_1_–*F_4_* and *D*_1_, the final similarity measure, is calculated based on the relationship:

(3) $$\begin{array}{c} \mathrm{sim}\left(x\right)=\frac{\sum_{i=1}^{n}\left|\left(a_{ib}-a_{iu}\right)\right|}{5}=\frac{\left|\mathrm{sim}\left(f_{1}\right)+\mathrm{sim}\left(f_{2}\right)+\mathrm{sim}\left(f_{3}\right)+\mathrm{sim}\left(f_{4}\right)+\mathrm{sim}\left(d_{1}\right)\right|}{5}\\ =\frac{\left|\left(a_{F1b}-a_{F1u}\right)+\left(a_{F2b}-a_{F2u}\right)+\left(a_{F3b}-a_{F3u}\right)+\left(a_{F4b}-a_{F4u}\right)+\left(a_{D1b}-a_{D1u}\right)\right|}{5} \end{array}$$

**Figure 4:**
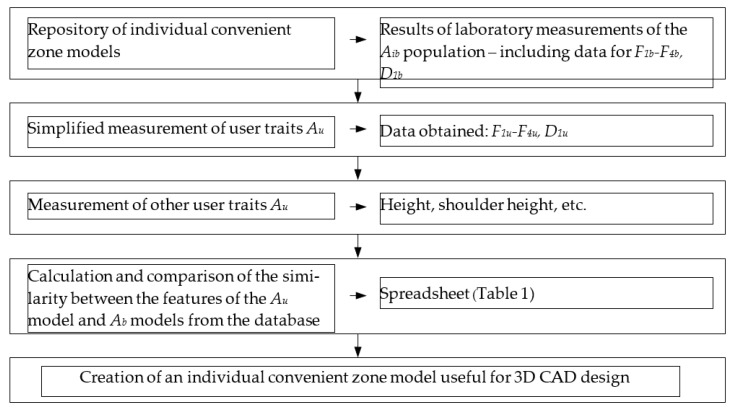
Algorithm for fitting the simplified *A_u_* model to a model from the *A_b_* database.

The algorithm for searching the repository of individual convenient zone models for the ICZ model that best fits the results of the simplified measurements is illustrated in Figure 4.

## 3. Verification of Methodology: Case Study

### 3.1. Problem Area 1: Creating a Database of Spatial and Strength Features. Problem Area 2: Identifying Spatial and Strength Characteristics of a Specific Human Being; Generating an Individual Convenient Zone Model

It is assumed that a suitable and comprehensive database of individual convenient zone models meets the criteria described in the standards, e.g., ISO 15537 [18]. Each model is detailed and marked as *A_b_*. The simplified data set, designated *A_u_*, consists of the following parameters: four upper limb strength values at specific points in the hand range space (*F*_1_, *F*_2_, *F*_3_, *F*_4_), and the anterior reach *D*_1_ to the handgrip axis (as shown in Figure 3). These parameters are relatively easy to measure or identify during the interview (body weight, height, and other body dimensions).

The person in our example was a 72-year-old senior with a body weight of 84 kg and an overall height of 175 cm (a shoulder height of 139 cm and an elbow height of 109 cm). Other parameters required for similarity computing were: Measured force *F*_1*u*_ = 71 N, *F*_2*u*_ = 57 N; *F*_3*u*_ = 146 N at a distance from the body axis *D*_1*u*_/2 = 34 cm, *F*_4*u*_ = 110 N, and measured hand range *D*_1*u*_ = 68 cm. All these parameters constitute the *A_u_* data set of a specific human, as mentioned earlier. The similarity between the *A_b_* ICZ models and *A_u_*, for the person in our example, is represented by the similarity value sim(*x*) = 0.085. Row 7 of Table 1 represents the best match between the five characteristics of the person *A_u_* and the attributes of the *A_b_* model chosen from the twelve characteristics in the database.

The best partial matching results for features *F*_1_ to *F*_4_ and *D*_1_ are highlighted in bold type in Table 1. This shows that these partial results may be better than the partial results of the chosen solution, item No. 7. As an example, the strength data *F*_1_ of the person in item No. 5 form a similarity with the *A_u_* score equal to sim(*f*_1_) = 0.064, and the scores in item No. 7 and *A_u_* form a similarity with greater (worse) variation equivalent to sim(*f*_1_) = 0.218. However, the overall fit for item No. 5 is worse than the overall result for sim(*x*) = 0.347, so this solution is rejected for the accepted criteria values.

Creating a comprehensive *A_b_* database is conducive to improving the matching of *A_b_* scores with *A_u_* and is also a factor in achieving closer sim(*x*) matches. However, even the database of 12 people used in our example permits the designer to use a dataset (*A_b_*_7_) of numerical and geometric 3D CAD data, analogous to that shown in Figure 1 and Figure 2, for the better customization of products. The resulting fit can be considered good: sim(*x*) = 0.085 for extreme values from 1 to 0.

### 3.2. Problem Area 3: Ergonomic Analysis of the Product

**Figure 5:**
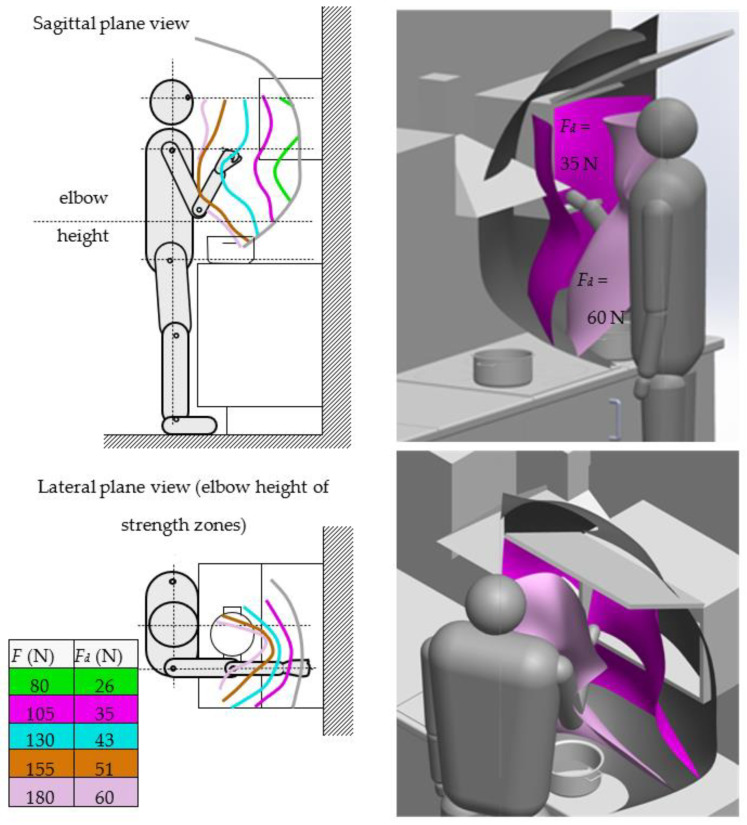
Visualization of the range zone and constant-strength space of the right limb during manipulation at the cooker and cabinets (two strength layers were used for testing) (*F*—measured strength; *F_d_*—permissible strength).

Figure 5 illustrates how the method can be used to analyze the accessibility of a kitchen for a senior citizen or person with disabilities. The choice of furniture is based on requirements, mainly derived from the end user’s demands and wishes [24,25,26]. However, in the furniture available on the market, these requirements are determined mainly by the subjective feelings of the customer. By using the method described, the design requirements resulting from the customer’s performance limitations can be identified based on a series of measurements, which are then processed into a form suitable for designing a customized kitchen. Therefore, identifying the customer’s unique characteristics is a crucial process, which may begin in the furniture shop at the point of sale. Designing a personalized kitchen using individual convenience zone (ICZ) information does not introduce significant changes. It consists of selecting multivariant modular furniture and creating a set of furniture adapted dimensionally and topologically to the requirements of even atypical customers. An example visualization of the verification and accuracy of the decision in terms of dimensions and topology of a furniture set is shown in Figure 5. The figures indicate the 3D location of the hand range and constant-strength zones. 

The analyzed constant-strength layers (e.g., *F* = 80, 105, 130, 155, 180 N) give the designer information about the individual’s strength capabilities. They enable the space to be designed in such a way as to ensure that the weight of objects to be moved, e.g., taken out of a kitchen cupboard, is not hazardous to a particular user. However, the *F*-force values measured in laboratory tests must be corrected (reduced in value) to an acceptable value of *F_d_* according to various practical aspects of work such as convenience, load, or load-holding time. In other words, *F_d_* force values are used to create convenient zone models in the database. This rescaling of the limit forces F to the allowable forces *F_d_* is proportional and does not change the graphical form of the 3D data obtained in the laboratory test. However, strength rescaling is not the subject of this paper; it is described in various works, e.g., [27]. The renderings (Figure 5, right-hand side) show the two chosen constant-strength layers, *F_d_* = 35 N and *F_d_* = 60 N. For example, a layer marked with a force value of *F_d_* = 10 N indicates that the lifting zone for objects with a force of 35 N should not be positioned further than the location of this layer. This area contains, e.g., the front shelf of a wall unit. Placing objects further from the body’s axis may cause discomfort or pose a health risk. Pots on standing cabinets can be safely lifted with a force of 60 N in the zone close to the body axis (light pink zone, *F_d_* = 60 N).

The availability of an extensive database (i.e., test results from a large number of people) of laboratory measurements facilitates their use in the selection of furniture for any user with individualized anthropometric (e.g., arm reach zones) and biomechanical (e.g., strength capabilities during lifting and carrying) characteristics.

## 4. Conclusions

Our proposed method is useful for the personalized design of the space surrounding a person. This method involves:

1 Quickly measuring just a few critical spatial and strength characteristics of a person and creating a simplified *A_u_* model of their individual convenient zone;
2 Selecting the best-fit and complete convenient zone model *A_u_* from the database based on the similarity criterion;
3 The selected model *A_u_* describes the “individual convenient zone” (ICZ) of a human subjected to rapid measurement and is helpful in product design.

The proposed method of matching the features of the *A_b_* laboratory test subjects to the *A_u_* user ensures that the design requirements are fulfilled appropriately, taking into account the features of the *A_u_* user that are easy to measure. However, some discrepancies may occur in *A_b_* relative to *A_u_* traits, even where an extensive database of *A_u_* individuals tested in the laboratory is available. These discrepancies are, as always, due to variations in anthropometric and biomechanical characteristics in the population, etc., and are subject to various ergonomic design guidelines. They may also be attributed to a lack of consideration of properties that are difficult or more complicated to examine, such as: The ratio between muscle mass and body fat mass; the effect of spinal cord damage height in disabled people; the associated strength capabilities of selected muscles, including the impact of dysfunction of, e.g., the shoulder joint, on strength performance among seniors.

Using an ICZ model, it is easy to analyze how a product fits into this zone at the design stage and thus positively influences its ergonomic quality. These parameters will serve to protect the customer from discomfort and overloading of the body when using products such as kitchen furniture. Individual customers cannot determine this data themselves as the data are not readily available in anthropometric atlases, biomechanical atlases, or the virtual anthropometric models currently used in CAD.

The application of ICZ effectively involves end-users in product design (participatory design). It enables product usability to be tailored precisely to the user (user-centered design). The ICZ modeling method described in this article may find broader applications beyond kitchen furniture design; it could be used in the design of workspaces and other similar areas where humans reside and perform manipulation activities. Human-systems inclusion requires that the automatic system has to take into account and adapt to all users whatever their cognitive state or disability.

## Figures and Tables

**Table 1:** Example of a worksheet to calculate the similarity between a simplified model of an individual convenient zone and a complete model from the database.

Features of Examined Persons *A_b_*							The Similarity between *a_u_* and *a_b_* [-] [1,0]					
Lp	*A_b_/* *a_b_*	*F*_1_*(*N)*/* *f*_1_ (–)	*F*_2_*(*N)*/* *f*_2_ (–)	*F*_3_ *(*N)*/* *f*_3_ (–)	*F*_4_ *(*N)*/* *f*_4_ (–)	*D*_1_ (cm)*/* *d*_1_ (–)	sim(*f*_1_)	sim(*f*_2_)	sim(*f*_3_)	sim(*f*_4_)	sim(*d*_1_)	sim(*x*)
1	*A_b_*/	62/	50/	125/	85/	71/	0.115	0.103	0.12	0.277	0.176	0.158
	*a_b_*	0.449	0.382	0.437	0.462	1						
2	*A_b_*/	82/	62/	154/	118/	54/	0.141	0.074	0.046	**0**	0.824	0.217
	*a_b_*	0.705	0.559	0.603	0.739	0						
3	*A_b_*/	64/	52/	170/	83/	66/	0.09	**0.073**	0.138	0.294	0.118	0.143
	*a_b_*	0.474	0.412	0.695	0.445	0.706						
4	*A_b_*/	63/	64/	90/	80/	67/	0.102	0.103	0.321	0.319	0.059	0.181
	*a_b_*	0.462	0.588	0.236	0.42	0.765						
5	*A_b_*/	66/	75/	94/	77/	55/	**0.064**	0.265	0.298	0.344	0.765	0.347
	*a_b_*	0.5	0.75	0.259	0.395	0.059						
6	*A_b_*/	27/	24/	49/	30/	70/	0.564	0.485	0.557	0.739	0.117	0.492
	*a_b_*	0	0	0	0	0.941						
**7**	***A_b_*** **/**	**88/**	**69/**	**150/**	**117/**	**68/**	**0.218**	**0.177**	**0.023**	**0.008**	**0**	**0.085**
	***a_b_***	**0.782**	**0.662**	**0.58**	**0.731**	**0.824**						
8	*A_b_*/	87/	86/	115/	105/	58/	0.205	0.427	0.178	0.109	0.589	0.302
	*a_b_*	0.769	0.912	0.379	0.63	0.235						
9	*A_b_*/	87/	79/	167/	99/	57/	0.205	0.324	0.121	0.159	0.648	0.291
	*a_b_*	0.769	0.809	0.678	0.58	0.176						
10	*A_b_*/	105/	92/	223/	149/	65/	0.436	0.515	0.443	0.261	0.177	0.366
	*a_b10_*	1	1	1	1	0.647						
11	*A_b_*/	81/	79/	102/	103/	68/	0.128	0.324	0.252	0.126	**0**	0.166
	*a_b_*	0.692	0.809	0.305	0.613	0.824						
12	*A_b_*/	57/	45/	141/	69/	67/	0.179	0.176	0.028	0.411	0.059	0.171
	*a_b_*	0.385	0.309	0.529	0.328	0.765						
	*A_b min_*	27	24	49	30	54						
	*A_b max_*	105	92	223	149	71						
User features *A_u_*												
		*F*_1_ (N)/ *f*_1_ (–)	*F*_2_ (N)/ *f*_2_ (–)	*F*_3_ (N)/ *f*_3_ (–)	*F*_4_ (N)/ *f*_4_ (–)	*D*_1_ (cm)/ *d*_1_ (–)						
*A_u_*/		71/	57/	146/	110/	68/						
*a_u_*		0.564	0.485	0.557	0.739	0.824						

Values highlighted in bold type are the best partial matching results (1—dissimilar, 0—similar).

## Data Availability

All data, models, and code generated or used during the study appear in the submitted article.
